# The Modification of Nitrogen to Modulate Perovskite for the Application of p-Type Transparent Conductive Oxides

**DOI:** 10.3390/molecules31020222

**Published:** 2026-01-08

**Authors:** Yunting Liang, Kaihua Li, Haixu Chen, Yinling Wang, Shasha Zheng, Liuyang Bai

**Affiliations:** 1 School of Energy Engineering, Huanghuai University, Zhumadian 463000, China; yicenli@foxmail.com (K.L.); chenhaixu@huanghuai.edu.cn (H.C.); wangyinling@huanghuai.edu.cn (Y.W.); zhengshasha@huanghuai.edu.cn (S.Z.); 2 Theoretical Materials Physics, Q-MAT, CESAM, University of Liège, B-4000 Liège, Belgium

**Keywords:** transparent conductive oxides, perovskite oxides, DFT, N-O ratio, transmission, conductivity

## Abstract

Due to the strong electronegativity of oxygen ions, the valence band maximum (VBM) that is derived from the O 2p orbital leads to strong localization, as well as further heavy hole mass and low hole mobility, which makes it extremely difficult to obtain high-conductivity p-type transparent conductive materials. Herein, we propose the strategy of multiple anions through the introduction of weaker electronegative nitrogen, in consideration of the delocalization on VBM, as well as the stability of octahedral anion cages. As such, first-principles calculations in the framework of density functional theory (DFT) are used for this work. Crystal structure prediction software USPEX (version 2023.0) was adopted to investigate the N-O appropriate ratio in CaTiO_3−x_N_x_ (0 ≤ x ≤ 1) to balance the high transmission of light and highly favorable dispersion at the VBM. Furthermore, the p-type TCO performance of CaTiO_3-x_N_x_ was evaluated based on the hole effective mass, hole mobility, and conductivity. The effectiveness of modulating p-type TCO through N-O multiple anions was also evaluated through defect formation energy and ionization energy. Ultimately, the construction of a CaTiO_3-x_N_x_/Si heterojunction and band alignment were considered for practical application. This approach attempts to boost the diversity of p-type perovskite-based TCOs and opens a new perspective for engineering and innovative material design for sustainable TCOs demand.

## 1. Introduction

In recent years, transparent conductive films have played a crucial role in various fields, such as solar cells, smart electronic products, liquid crystal displays, and conductive glass curtain walls, owing to their unique photoelectric properties. It is estimated that the global market size of transparent conductive films will reach 1.27 billion US dollars by 2029 [1]. Among them, n-type ITO (indium-doped tin oxide) has occupied 90% of the transparent conductive film market, owing to its excellent photoelectric properties, while the share of p-type transparent conductive oxides is relatively rare. This situation is mainly caused by the strong localization of the valence band top of p-type oxides, which leads to a low hole conductivity and, thus, severely limits the application and development of high-performance p-type transparent conductive oxides in transparent electronic devices [2]. Therefore, the exploitation of high-performance p-type transparent conductive oxides and their application in heterojunction devices are of great significance to research.

Transparent conductive oxides (TCOs) need to meet two key technical requirements: one is spectral transmittance [3]. To fully utilize sunlight, TCO-coated glass needs to have a transmittance of ≥80%. The other is electrical conductivity. TCO conductive films improve the film’s conductivity through doping, with minimum resistivity reaching the 10^−5^ Ω·cm level. Currently, n-type electron-conducting TCOs have been commercialized, but p-type TCOs are still in the early stages. The key challenge for p-type TCOs lies in how to reduce the strong localization of the valence band formed by O 2p states in metal oxides [4].

Over the past decades, continuous efforts have been made to try to solve this problem. In 1997, Kawazoe et al. first reported the p-type transparent conductive properties of CuAlO_2_ with a delafossite structure, and they proposed a novel approach—the Chemical Modification of the Valence Band (CMVB) theory [5]. This theory creatively proposes the use of cations with a full shell d^10^, as well as their energy levels equivalent to the O 2p levels, and then cation and anion form anti-bonding states through orbital hybridization, thereby weakening the localization effect of the holes near the valence band edges. This ultimately achieves the goal of improving p-type conductivity. Based on this, numerous delafossite-structured AMO_2_ materials are widely studied in p-type TCO films [6,7], e.g., Cu-based CuAlO_2_, CuCrO_2_, CuScO_2_, CuYO_2_, and CuGaO_2_, as well as Ag-based AgCrO_2_, AgGaO_2_, and AgCoO_2_. Limited by the existence of strongly electronegative oxygen ions, the room-temperature p-type conductivity of CuAlO_2_ only reaches up to 1 S cm^−1^ to date, which lags far behind that of the industrial standard n-type transparent conducting oxides [8]. Therefore, there is still an urgent demand to find an appropriate path to achieve high-performance p-type TCOs.

Perovskite oxides gradually came into the view of researchers, owing to their natural high light transmittance from a wide optical gap (>3.0 eV), adjustable band edge from doping, and excellent electrical properties from their extraordinary crystal structures [9]. Kim et al. reported that La-doped BaSnO_3_ obtained high mobility at room temperature while retaining its optical transparency [10]. In single crystals, the mobility reached 320 cm^2^V^−1^s^−1^ at a doping level of 8 × 10^19^ cm^−3^. In epitaxial films, the maximum mobility was 70 cm^2^V^−1^s^−1^ at a doping level of 4.4 × 10^20^ cm^−3^. From this perspective, it reveals the excellent electrical potential of perovskite oxides as TCOs. Sr-doped LaCrO_3_ with 50% Sr on the La site, arguably forming a mixed cation perovskite, resulted in p-type conductivity of 50 S cm^−1^, but at the cost of a 50% loss in transmission [11]. The p-type K-doped BaSnO_3_ showed a hole mobility of 0.30 cm^2^V^−1^s^−1^ at a carrier concentration of 10^13^ cm^−3^, giving low overall conductivity [12]. The rhombohedral double perovskite Ba_2_BiTaO_6_ with K on the Ba site was demonstrated to display a hole mobility of 30 cm^2^V^−1^s^−1^, but with an extremely low carrier concentration (10^14^ cm^−3^) and, therefore, low conductivity [13]. The application of perovskite as a p-type TCO is still in its initial stage, and the optimization of the modulation technology is still ongoing.

Recently, the modulation strategy of multiple anions played an important role in the physical and chemical properties of materials. Kageyama et al. [14] mixed other anions in traditional single-anion compounds and changed the number, position, and size of the substituted anions to transform energy level splitting under the crystal field; this was done to further the control of the energy band, and ultimately develop new applications of traditional materials, such as photocatalytic applications (Zr_2_ON_2_) (N_1-x_O_x_) [15], battery applications (LiFeSO_4_F) [16], polychromatic applications (Ca_3_ReO_5_Cl_2_) [17], superconductivity applications (Sr_2_CuO_2_Cl_2_) [18], and so on. At present, research that is based on perovskite-type TCOs mostly focuses on the modulation of metal cations [10,12]; however, there is little research on strategies of multiple anions to design p-type TCOs. In consideration of the prominent effect of multiple anions on the electrical properties of materials, this work will carry out the introduction of weaker electronegative nitrogen to exploit perovskite as a candidate for p-type TCOs.

This work was conducted based on parent CaTiO_3_ with a natural wide gap (>3.0 eV) and lead-free resource. The paper is organized as follows. First, the O-N ratio in CaTiO_3-x_N_x_ (0 ≤ x ≤ 1) is searched and discussed, focusing on their crystal structures, band structures, and optical properties. Second, the performance of CaTiO_3-x_N_x_ is evaluated as a p-type TCO application, focusing on elastic constants, deformation potential, effective mass, mobility, and conductivity. The effectiveness of modulating p-type TCOs with a N_O_ defect in CaTiO_3_ is evaluated through defect formation and ionization energy. In conclusion, the photovoltaic heterojunction is constructed, and the band alignment is carried out based on p-type CaTiO_3-x_N_x_ on a Si substrate.

## 2. Results and Discussion

### 2.1. Search for O-N Ratio in CaTiO_3-x_N_x_ (0 ≤ x ≤ 1)

**Figure 1:**
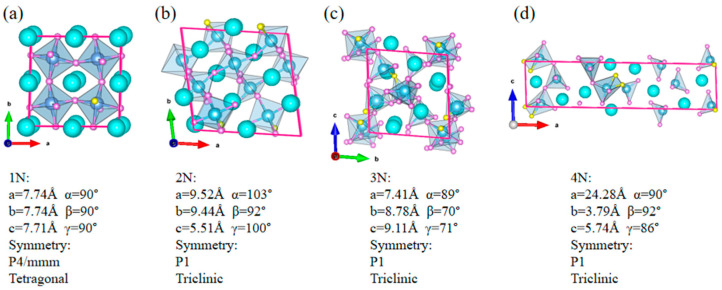
The crystal structure of a CaTiO_3-x_N_x_ (0 ≤ x ≤ 1) supercell, searched by USPEX software: (**a**) 1N (x value: 0.025), (**b**) 2N (0.05), (**c**) 3N (0.075), and (**d**) 4N (0.1).

Using the crystal structure prediction software, USPEX, the O-N ratio in CaTiO_3-x_N_x_ (0 ≤ x ≤ 1) was predicted through variational control. 1N, 2N, 3N, and 4N were sequentially doped into the parent phase CaTiO_3_ (CTO) in a 2 × 2 × 2 supercell (40 atoms), with the corresponding x values of 0.025, 0.05, 0.075, and 0.1, respectively. After global searching, stable crystal structures were obtained at different doping concentrations and are shown in Figure 1. In Figure 1a, after replacing O with 1/24 N in the 40-atom supercell, the crystal structure transforms from the orthorhombic phase of the parent phase CaTiO_3_ to a more symmetrical tetragonal phase (space group: P4/mmm). As the number of N continues to increase, the symmetry of the crystal structure decreases to its minimum (space group: P1), making it difficult to stably exist in terms of energy. Therefore, in CaTiO_3-x_N_x_ (0 ≤ x ≤ 1), the thermodynamic stability of the crystal is optimal when the N concentration is 0.025, which is the best doping concentration in the parent phase CaTiO_3_.

**Figure 2:**
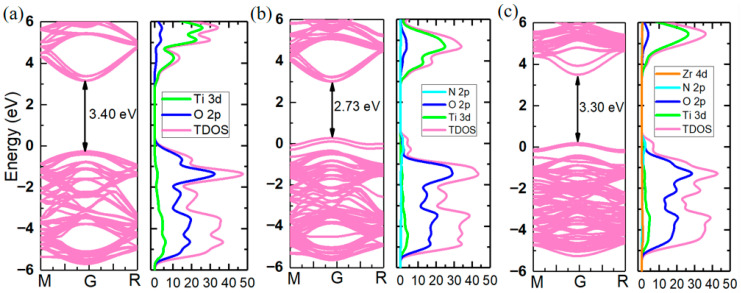
Band structure and density of states through HSE06 hybrid functional calculation: (**a**) CaTiO_3_, (**b**) CaTiO_2.975_N_0.025_, and (**c**) CaTi_2.975_Zr_0.025_O_2.975_N_0.025_.

Subsequently, the electronic structure of the CaTiO_2.975_N_0.025_ supercell (CTON) was calculated using HSE06 hybrid functional, and the results are shown in Figure 2b. Compared to the CaTiO_3_ band structure, where the valence band top is mainly contributed by O 2p orbitals (Figure 2a), in the CTON system, two impurity energy levels are introduced at the valence band top due to the substitution of weak electronegative N doping, resulting in a decrease in the optical gap width from 3.4 eV to 2.73 eV. Since N doping only acts on the valence band, the position of the conduction band bottom in the CTON system remains unchanged at around 3.0 eV. Considering the high transparency of transparent conductive oxides, candidate materials need to have a band width that is greater than 3.0 eV. Therefore, further substitution is needed in the CTON system to increase the bandwidth. As the conduction band in the CaTiO_3_ band structure is mainly contributed by Ti 3d orbitals, one Zr atom with a higher orbital level is selected for the substitution on one Ti site, aiming to promote the upward lifting of the conduction band and ultimately increase the band width of CTON to the ideal range.

**Figure 3:**
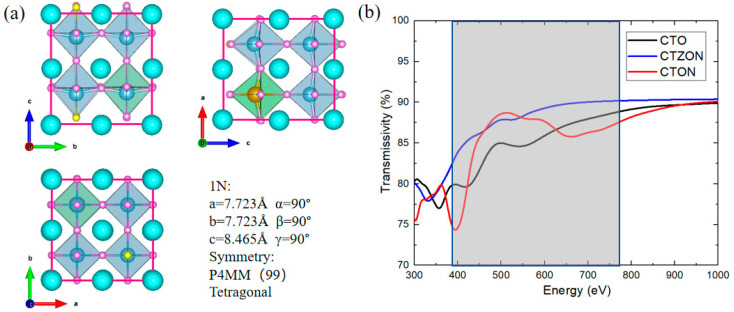
(**a**) Crystal structure of CaTi_2.975_Zr_0.025_O_2.975_N_0.025_-doped system (CTZON), (**b**) comparison of transmittance of three types of thin-film materials.

The crystal structure of the CaTi_2.975_Zr_0.025_O_2.975_N_0.025_-doped system (CTZON) is shown in Figure 3a. The Zr cation has a bigger ionic radius (0.72 Å) than the Ti cation (0.605 Å), under the VI coordination number. Therefore, the introduction of one Zr cation brings out a degree of octahedron distortion after fully relaxing. Owing to the tolerance of the octahedral oxygen cage, after Zr substitution, the crystal structure of the system remains tetragonal, and the symmetry slightly decreases to P4MM. The electronic structure is shown in Figure 2c, and occurred after the HSE06 hybrid functional calculation was completed. Due to the substitution of one Zr atom for one Ti site, the conduction band position is shifted to a higher energy level, resulting in an expansion of the band width from 2.73 eV to 3.30 eV, making the N-doped system meet the high transparency requirements.

The optical properties of materials can be obtained from the frequency-dependent complex dielectric constant ε(ω) through VASPKIT (version 1.5.0) [19], and the calculation Formula (1) is as follows:

(1) $$\varepsilon (\omega )=\varepsilon_{1}(\omega )+\varepsilon_{2}(\omega )$$

Among them, $\varepsilon_{1}(\omega )$ and $\varepsilon_{2}(\omega )$ are, respectively, the real and imaginary parts of the dielectric constant, and ω is the photon frequency.

The transmittance T(ω) of a material is related to its reflectivity R(ω), absorption coefficient β(ω), and thickness d in the incident direction. Formula (2) is as follows:

(2) $$T(\omega )={\left[1-R(\omega )\right]}^{2}exp\left[-\beta (\omega )d\right]$$

The calculation Formulas (3) and (4) for reflectivity and the absorption coefficient are as follows:

(3) $$R(\omega )=\frac{{\left[n(\omega )-1\right]}^{2}+{k(\omega )}^{2}}{{\left[n(\omega )+1\right]}^{2}+{k(\omega )}^{2}}$$

(4) $$\beta (\omega )=\frac{4\pi \omega k(\omega )}{c}$$

In the formulas for reflectivity and the absorption coefficient, n(ω) and k(ω) are for the refractive index and extinction coefficient, respectively. The calculation Formulas (5) and (6) are for the relationship between the real and imaginary parts of the dielectric constant and are as follows:

(5) $$n(\omega )=\sqrt{\frac{\sqrt{\varepsilon_{1}{\left(\omega \right)}^{2}+\varepsilon_{2}{\left(\omega \right)}^{2}}+\varepsilon_{1}(\omega )}{2}}$$

(6) $$k(\omega )=\sqrt{\frac{\sqrt{\varepsilon_{1}{\left(\omega \right)}^{2}+\varepsilon_{2}{\left(\omega \right)}^{2}}-\varepsilon_{1}(\omega )}{2}}$$

After a series of calculations of optical parameters, the transmittance of the doped film is shown in Figure 3b. The transmittance of CTZON is significantly better than that of the CTO and CTON systems in the visible light range of 390–780 nm, and the transmittance of the CTZON system reaches up to 90% in the visible light range.

### 2.2. Performance Evaluation of CaTiO_3-x_N_x_ (0 ≤ x ≤ 1) as p-Type TCO Applications

Adopting an approximate method based on deformation potential theory to study the p-type transport characteristics of CTZON, the mobility formula is as follows [20]:

(7) $$\mu =\frac{{\left(8\pi \right)}^{1/2}{ћ}^{4}ec_{ii}}{3{\left(m^{*}\right)}^{5/2}{\left(k_{B}T\right)}^{3/2}{E_{1}}^{2}}$$

Among them, ћ is the reduced Planck constant, e is the electron charge, $c_{ii}=V_{0}(\frac{\partial^{2}E}{\partial V^{2}})$ is the elastic constant, V_0_ is the lattice volume, m* is the effective mass of the carrier (electrons for the bottom of the conduction band and holes for the top of the valence band), k_B_ is the Boltzmann constant, T is the temperature of 300 K, and $E_{1}=\frac{{∆E}_{BM}}{∆V/V_{0}}$ is the deformation potential, defined by the change in band edge energy divided by the change in volume. The effective mass m* of the valence band holes in the researched system is obtained using EMC software (version 1.0) based on the finite difference method.

The elastic constants, deformation potential, effective mass, and mobility obtained through calculations are shown in Table 1. Due to the strong oxygen localization in CTO, the dispersed morphology of the valence band top produces a hole effective mass of 9.72 m_0_, which is not conducive to the efficient migration of hole carriers. This conclusion is close to the hole effective mass of 9.8 m_0_ that was reported by Wunderlich et al. for SrTiO_3_ at the G point [21]. After N doping, the effective mass of the holes in CTON is significantly improved compared to CTO, with an effective mass reduction to 2.09 m_0_. This is mainly due to the formation of two new, sharper impurity energy levels from the weaker localization at the top of the valence band after N doping. However, Zhong et al. found that the effective mass of holes needs to be reduced to below 1.5 m_0_ in order to facilitate efficient transport of hole carriers [22]. In the CTZON system, one Zr atom with strong metallicity further optimizes the atomic bonding in the doping system, thereby improving the local morphology of the valence band top. After calculation, a hole effective mass of 1.24 m_0_ is obtained, indicating that the doping ratio in CTZON has achieved the goal of efficient modulation of CaTiO_3_ for p-type transparent conductive oxide applications.

After calculating the mobility, it was found that compared to the mobility of 1.49 cm^2^/v/s in the CTO system, the CTON system achieves a mobility of 39.73 cm^2^/v/s, which is 10 times higher; the mobility of the CTZON system is 158.73 cm^2^/v/s, which is 10^2^ times higher and significantly improves the transport ability of the valence band top holes. In a supercell with 40 atoms, the doping concentration of one N atom was calculated to be 5.51 × 10^19^/cm^3^. The conductivity of the system was calculated using the formula σ = neμ (n-doping concentration, e-elementary charge, and μ-mobility) and the results as shown in Table 1. Compared to the weak conductivity of 13 S/cm in the CTO system, the CTON system achieves a conductivity of 349 S/cm, while the conductivity of the CTZON system is greatly improved, reaching 1397 S/cm, which is far superior to the conductivity level of traditional p-type delafossite-based transparent conductive oxides [8].

The effectiveness of modulating p-type TCO with a N_O_ defect and the compensation effect of a V_O_ defect on p-type doping can be evaluated by the formation energy of defects [23]. The calculation formula for defect formation energy is as follows:

(8) $${\Delta E}_{f}=E_{tot}-E_{pure}+\sum n_{i}\mu_{i}+qE_{f}+∆V$$

In the equation, E_tot_ represents the total energy of one supercell containing a defect D with q charge, E_pure_ is the total energy of a perfect system of the same size, n_i_ is for the change in the number of atoms in the system before and after introducing the defect (adding atoms to the system (n_i_ < 0) and removing atoms from the system (n_i_ > 0)), μ_i_ is for the atomic chemical potential of the constituent elements, and the Fermi level E_f_ refers to the energy position E_V_ (Perfect) of VBM in the perfect system.

We introduced a correction term ∆V to correct the electrostatic potential difference between the defective supercell and perfect supercell: ΔV = V_ave_ (D, q) − V_ave_ (perfect, 0), where V_ave_ (D, q) and V_ave_ (perfect, 0) are the average electrostatic potentials of the defective supercell and perfect supercell, respectively.

The calculation found that the formation energy of the N_O_ defect is −2.62 eV, indicating that N-substituted O is prone to occur and the defect can exist stably. The formation energy of the V_O_ defect is 2.07 eV, testifying that due to the stabilizing effect of TiO_6_ octahedral oxygen cages in the CTO parent phase, the V_O_ defect is not easily generated. Therefore, the compensation effect of the V_O_ defect on the N-substituted modulation of CaTiO_3_ for p-type TCO is not prominent. The stability and effectiveness of N-substituted modulation are ensured through the calculation of formation energy.

Further research was conducted on the ionization energy of the defects in the CTZON system to ensure that the acceptor level position of the N_O_ defects was shallow for ionization. Using a hydrogen-like model, the ionization energy of the material was calculated based on the effective mass of carriers and the relative dielectric constant [24]. The ionization energy is defined as the energy difference between the acceptor/donor energy levels and the VBM state. The formula for calculating the ionization energy of one single hole state is as follows:

(9) $$E_{1}=\frac{e^{4}m^{*}}{({4\pi \varepsilon_{0})}^{2}m_{0}}\times \frac{1}{\varepsilon_{2}}=13.6\frac{m^{*}}{m_{0}\varepsilon^{2}}$$

In this equation, 13.6 eV is the ground state ionization energy of hydrogen atoms, m* is the effective mass of the system, m_0_ is the effective mass of the elementary charge, and ε is the relative dielectric constant. After calculating the relative permittivity through VASP software (version 5.4.4), it is known that the ε of the CTO system is 10.81 and the ε of the CTZON system is 11.20. Based on the calculated effective mass of the holes in Table 1, here, the ionization energies at the valence band tops of CTO and CTZON are 1.13 eV and 0.13 eV, respectively. N-doping effectively reduces the ionization energy of the valence band top and facilitates the formation of p-type hole carriers.

### 2.3. Photovoltaic Heterostructure Construction and Band Alignment Based on p-Type CaTiO_3-x_N_x_

**Figure 4:**
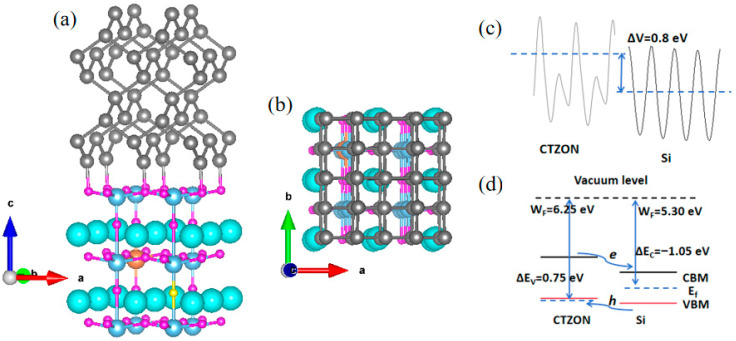
(**a**,**b**) show the CTZON/Si heterostructure, (**c**) the average electrostatic potential difference between CTZON and Si, and (**d**) the band edge offset and band alignment type of CTZON/Si.

The interface modeling adopts the superlattice method with periodically repeating layers, and the superlattice based on p-type CTZON and Si is constructed through Material Studio software (version 2017) for the application of p-i photovoltaic heterostructure. The (001) crystal plane of CTZON (a = b = 7.723 Å, c = 20.251 Å, α = β = γ = 90°), and the (011) crystal plane of Si (a = b = 7.734 Å, c = 19.570 Å, α = β = γ = 90°) were selected for interfacial matching, and the lattice mismatch was calculated to be 0.14%, indicating that the two have good lattice matching abilities and can effectively avoid internal stress at the interface. The CTZON/Si heterostructure is shown in Figure 4a,b. In Figure 4a, Si atoms tend to bond with O atoms in the TiO_2_ layer, effectively reducing the occurrence of oxygen vacancies at the interface and eliminating the negative impact on the p-type TCO properties of N-doped CaTiO_3_. From Figure 4b, it can be seen that the Si (011) interface matches well with the CTZON (001) interface.

To investigate the types of band alignment of the CTZON/Si heterostructure, the average electrostatic potential alignment method was used for research [25,26]. First, the average electrostatic potential of two materials based on the vacuum energy levels was obtained, as shown in Figure 4c. Considering the Fermi levels of the two materials, the work functions of the two materials were obtained, as shown in Figure 4d. It can be seen that the difference in work functions between the two materials is 0.95 eV. According to the positions of the valence band top and conduction band bottom of CTZON and Si, the valence band deviation of the two materials was calculated using ΔE_VBM_ = −0.2 eV, and the conduction band deviation ΔE_CBM_ = −2.0 eV. Furthermore, it can be inferred that the band offset of CTZON and Si is ΔE_V_ = 0.75 eV, and the band offset is ΔE_C_ = −1.05 eV. From the band offsets, it can be concluded that the CTZON/Si heterostructure belongs to the type II stagger, which can effectively promote electron–hole spatial separation and the efficient transport of hole carriers.

## 3. Methods

Theoretical simulation calculations were carried out using the Vienna Ab Initio Simulation Package (VASP) [27,28], while the projector augmented wave (PAW) method was used to describe the ionic potentials, including the effect of the core electrons [29,30]. For calculation accuracy, the Brillouin Zone (BZ) summation was calculated with Monkhorst–Pack k-point intervals that were limited below 0.04 Å^−1^ for the supercells [20]. A plane-wave cutoff energy of 520 eV was employed in all calculations. All obtained structures were geometrically fully relaxed until the force convergence on each ion was reduced below 0.01 eV Å^−1^ [31]. We adopted a convergence criterion of 10^−6^ eV, which is adequate for electronic self-consistent iteration. For the calculations of band structures, we used the HSE06 functional to achieve more accurate values of band gaps [32]. In this work, the Perdew–Burke–Ernzerhof (PBE) exchange–correlation (XC) functionals [33,34] were adopted to calculate the electronic properties.

Based on the genetic evolution algorithm with the principle of energy minimization, the universal structure predictor (USPEX) [35,36] was adopted to predict stable or metastable structures for any given composition. The prediction process was as follows: in the first generation crystal structure prediction, 200 initial structures were randomly generated after spatial symmetry operation; after comparing the enthalpy of relaxation structure formation, the most stable and metastable structures were selected and entered the next generation cycle; in each subsequent generation, an additional 60 crystal structures were obtained, with variable values of 50% inherited, 20% random, 20% soft mode mutation, and 10% lattice mutation. Continuous cycling was essential until the most stable component structure remained constant for the subsequent 20 generations to ensure global equilibrium. Then, the search cycle ended.

## 4. Conclusions

In this study, we used the strategy of multiple anions to modulate multifunctional perovskite oxides for the application of p-type TCOs against strong localization from the O 2p orbital at the VBM. From the enthalpy of formation, USPEX software finds that the N concentration with x = 0.025 in CaTiO_3-x_N_x_ could maintain the stability of octahedral anion cages without the severe destruction of symmetry. Employing the HSE06 hybrid functional, it was predicted that the optical gap in the CTON supercell would slightly decrease to 2.73 eV. Furthermore, substituting one Zr atom with a higher orbital level at the Ti site was selected to elevate the conduction band minimum and, consequently, expand the gap width to 3.30 eV. Subsequently, the transmittance of CTZON was confirmed to reach up to 90% in the visible light range of 390–780 nm. After calculating electrical performance, the CTZON compound contributes 1.24 m_0_ of hole effective mass, 158.73 cm^2^/v/s of hole mobility, and 1397 S/cm of hole conductivity. Meanwhile, the formation energy of the N_O_ defect with −2.62 eV estimates the effectiveness of modulating a p-type TCO through N-O multiple anions. Furthermore, the ionization energy of the N_O_ defect, with 0.13 eV, confirmed the shallow acceptor energy for facilitating the formation of p-type hole carriers. Additionally, the (001)CTZON/(011)Si heterojunction was constructed with a 0.14% lattice mismatch, and the band alignment demonstrated that it belongs to a type II staggered heterojunction, which can effectively promote electron–hole spatial separation and the efficient transport of hole carriers. This work provides fundamental insights into the design of high-performance p-type transparent materials, and it is supposed to enrich the diversity of alternative perovskite-based TCOs.

## Figures and Tables

**Table 1:** Elastic constants (C), valence band deformation potential (E_v_), effective hole mass (m*), hole mobility (μ), and conductivity (σ) of three materials.

	C (eV/Å^3^)	E_v_ (eV)	m* (m_0_)	μ (cm^2^/v/s)	σ (S/cm)
CTO	0.55	6.00	9.72	1.49	13
CTON	0.83	9.79	2.09	39.73	349
CTZON	0.75	8.97	1.24	158.72	1397

## Data Availability

The original contributions presented in this study are included in the article. Further inquiries can be directed to the corresponding authors.
